# Effect of College Students’ Academic Stress on Anxiety Under the Background of the Normalization of COVID-19 Pandemic: The Mediating and Moderating Effects of Psychological Capital

**DOI:** 10.3389/fpsyg.2022.880179

**Published:** 2022-04-13

**Authors:** Yong Yang, Pingzhan Yang

**Affiliations:** ^1^College of Art, Hunan City University, Yiyang, China; ^2^School of Educational Science, Hunan Normal University, Changsha, China

**Keywords:** normalization of COVID-19 pandemic, academic stress, psychological capital, anxiety, college students

## Abstract

Based on the background of the continuous development of COVID-19 pandemic, the effect of academic stress on anxiety of college students, as well as the mediating and moderating role of psychological capital are discussed, so as to provide intervention measures for reducing the academic stress and anxiety level of college students during the pandemic. The study used the Academic Stress Scale, the Psychological Capital Scale and the Anxiety Scale to conduct a questionnaire survey on 280 college students in five colleges and universities in Northern Hunan, and obtained 229 valid questionnaires. The data analysis results show that there are differences in academic stress between different genders, and differences in the variable of psychological capital among college students of different grades; Whether he is a student leader and whether he has won a scholarship, the difference of this factor in the three variables of academic stress, psychological capital and anxiety.is not statistically significant. The results of this study showed that psychological capital significantly negatively predicted anxiety (β = −0.602, t = −9.702, *p* < 0.001), academic stress significantly positively predicted anxiety (β = 0.247, t = 5.462, p < 0.001), psychological capital played a partial mediating role between academic stress and anxiety, and psychological capital also had a certain moderating role between academic stress and anxiety (β = −0.15, t = −4.51, *p* < 0.001). The conclusion of the study is that in the context of the continuous development of COVID-19 pandemic, positive psychological capital can effectively reduce the anxiety caused by academic stress. This result suggests that the positive psychological capital state of college students should be improved, which can effectively relieve pressure and reduce anxiety.

## Introduction

In December 2019, the outbreak of COVID-19 brought an unprecedented shock to education. In order to effectively stop the spread of the pandemic to schools, the Ministry of Education of China has requested that colleges and universities at all levels across the country delay the start of school in the spring of 2020. During this period, college students lacked campus learning activities, interpersonal communication and physical fitness activities, and the overloaded pandemic information and public opinion also had a certain negative impact on the psychology of college students. Almost all colleges and universities in China adopt the online teaching model for the spring semester. The large amount of teaching information and the new teaching model have exacerbated the anxiety and negative emotions of college students. In the fall of 2021, many colleges and universities still delayed the start of the semester by 2–5 weeks. During the normalization of the pandemic, in order to better protect the safety of college students, many university campuses have implemented the management model of “not going out of school unless necessary.” Students going out of the campus need to go through the counselor approval mechanism. Students’ going out behaviors such as traveling, going out of the province and even going home from the city are affected to varying degrees. Due to the impact of the pandemic, it has become normal for junior and senior students in many colleges and universities in China to postpone, cancel and change the form of social practice activities, graduation practice and professional practice. In addition, many senior students are facing severe challenges in the employment situation. On the premise of not being fully prepared, they choose postgraduate entrance examination, civil service examination and other ways to alleviate the employment pressure. These changes in learning styles and lifestyles have brought varying degrees of pressure to college students and led to varying degrees of anxiety.

Stress is a subjective experience generated by individuals on external events and a cognitive and behavioral experience process formed by individuals facing the social external environment (He, 2017). When an individual’s ability is challenged and he feels stimuli and threats, stress will occur, leading to various emotional reactions both physiologically and psychologically, among which anxiety, tension, and frustration are the most common experiences (Liao, 2017). Some scholars have found that academic stress is common among college students, it is even greater than that in senior high school, which has become the greatest pressure faced by college students (Xu, 2011). If the academic pressure of college students is high, it will affect the physical and mental health and emotional state of students to varying degrees, and is not conducive to the development of college students and their healthy growth (Li and Tian, 2021). The 2014 “China Education Development Report” clearly pointed out that the main reason for students’ suicide is excessive learning pressure. A series of serious consequences caused by heavy academic pressure have attracted scholars’ attention to the academic pressure of college students. Especially in the case of the continuous spread of the COVID-19 pandemic, how to effectively relieve the learning pressure of college students, reduce the anxiety level of college students, and promote the physical and mental health of college students through scientific and reasonable methods has become a research focus of scholars.

In the previous research, the impairment of physical and mental health caused by stress has been fully discussed. In the research field of stress relief, many scholars have noticed that individual positive psychological capital can effectively alleviate the anxiety caused by stress. Studies have shown that the more resilient, optimistic and positive psychological capital college students have, the lower their anxiety level is.

As for the influencing factors of College Students’ academic pressure, previous studies mostly start with the specific methods of pressure relief, but few studies discuss it from the perspective of the moderating effect of college students’ psychological capital. Research on the impact of COVID-19 on mental health is also mainly focused on medical staff, patients and their families, and there is a lack of research on the group of college students. At the same time, the normalized development of the pandemic in China and the measures to control the pandemic, as well as the serious development of the pandemic situation in foreign countries, and the increasing number of infections have brought great threat and impact to the study abroad of college students. In 2022, the number of college graduates in China exceeded 10 million for the first time, reaching a new record. The record number of graduates is superimposed on the downward pressure of the economy. The continuation of the pandemic has exacerbated the severity and complexity of the employment situation and brought great anxiety and pressure to college students about to graduate. Based on this, this paper relies on the relevant research results of college students’ academic stress, psychological capital and anxiety level, and analyzes the relationship between the academic stress, psychological capital and anxiety of college students under the background of the continuous spread of the pandemic, and whether psychological capital has a certain moderating role in the impact of academic pressure on anxiety, in the state of large-scale public health emergencies, it is of great practical significance to provide empirical research basis for relieving college students’ academic stress and improving the mental health level of this group.

## Theoretical Basis

### Academic Stress

Stress is generated by the interaction between individual and external environment. When individual’s ability is challenged and he feels stimulation and threat, it will produce various physiological and psychological emotional reactions. It is a relationship between human and specific environment. When individuals feel pressure, their perception and evaluation of the situation will produce psychological experience and emotional experience, among which anxiety, tension and frustration are the most common experience.

Regarding the definition of academic stress, there is no unified definition in the literature at home and abroad. The World Health Organization defines it as “some students understand some events encountered in the learning process as a threat to themselves, and then lead to a series of adverse reactions (such as worry, anxiety, or fear).” Lazarus (1990) believes that academic stress refers to the pressure that students feel in various disciplines in the process of learning. In the Dictionary of Psychology, Lin Chongde and others defined academic stress as: “The psychological burden and nervous reaction caused by learning, which is from the influence of the external environment and students’ self-expectation (Lin et al., 2004).” Based on the above views, academic stress can be defined as: in the process of learning, students’ nervous response to external stimuli is the result of the interaction between individuals and their surrounding environment. It is not only caused by external stimuli, but also depends on individuals’ cognition and evaluation.

In the previous research fields related to academic stress, the research contents mainly include the relationship between well-being and academic stress, and the regulation of college students’ academic stress. Based on the development background of the COVID-19 pandemic, the paper takes the academic stress of college students as an independent variable, and analyzes whether the academic stress has an impact on anxiety in a situation where the learning environment and learning method have changed significantly compared with the past, and whether positive psychological capital has a certain moderating effect and can effectively reduce anxiety, and further expands the research scope of academic stress.

### Psychological Capital

Psychological capital first appeared in the fields of economics and management. Goldsmith and others defined psychological capital as personality characteristics that can affect individual productivity, such as personal control point and self-esteem. These characteristics can affect an individual’s work motivation level and work attitude. Seligman (2002) expanded the objects of psychological capital and believed that those psychological factors that can affect individual positive psychological behavior can be regarded as psychological capital. Until 2005, Luthans clearly defined psychological capital as “the core psychological element of an individual’s general positivity, which is embodied in the psychological state in line with the standard of positive organizational behavior”, and then this definition was adopted by studies at home and abroad.

Since the birth of psychological capital theory, it has been widely concerned by researchers at home and abroad. Based on the existing literature, people find that the research on psychological capital at home and abroad mainly focuses on the intervention of psychological capital and the relationship between psychological capital and related variables. As a psychological state, psychological capital has certain plasticity. Luthans et al. (2005) first put forward the intervention theory of psychological capital and advocated the development of psychological capital from four different dimensions: self-efficacy, optimism, hope, and resilience. As for the theoretical structure of psychological capital, the two-dimensional theory represented by Goldsmith and others was first put forward, it is considered that two elements constitute psychological capital, that is, self-esteem and personal self-control point.Subsequently, Luthans and Youssef (2004) proposed that psychological capital is composed of three positive factors: resilience, optimism and hope, namely the three-dimensional theory; Until 2006, Luthans found that another positive psychological quality, namely self-efficacy, also met the standard category of psychological capital, so he put forward the four-dimensional theory, that is, the four positive psychological states of resilience, optimism, hope, and self-efficacy constitute psychological capital. Later, some researchers put forward the multi-dimensional theory, which holds that as long as the psychological factors that conform to and can promote the individual’s the individual’s positive psychological state and positive organizational behavior can be included in the psychological capital.

The current research of domestic and foreign scholars mostly follow the four-dimensional theory proposed by Luthans, that is, psychological capital includes four positive factors of resilience, optimism, hope, and self-efficacy. Its specific performance is: the individual’s confidence to achieve goals in a certain field (self-efficacy) and has ability to effectively complete tasks; Individuals have an optimistic interpretation style, can make positive attribution when encountering difficulties and setbacks, and maintain a positive attitude towards life (optimism); Adhere to the goal and adjust the path in time for success (hope); Ability to persevere, grow fast and face it with positive behavior in the face of setbacks and adversity (resilience). Xia (2011) and Yang (2014) both found through empirical research that psychological capital has a significant moderating effect on the pressure of teachers or company employees, and has a significant impact on occupational stress and occupational well-being. Individuals who have higher levels of psychological capital are more likely to relieve occupational stress and less likely to experience job burnout. Zhang’s (2013) research shows that psychological capital plays a partial mediating role in work stress response and stressful work sources, and psychological capital is also used as a moderating variable, and the study shows that the relationship between dependent variables and independent variables can be moderated by psychological capital. The dynamic effect model of psychological capital is proposed by Wang (2007), that is, the relationship between psychological capital and variables is not only a linear relationship, but may interact with each other. This research paradigm is still in the stage of theoretical hypothesis and needs to be tested by empirical research.

With the rapid development of positive psychology and positive organizational behavior, psychological capital, an internal personal resource for development, has attracted much attention in the research community. It is believed that psychological capital can effectively promote occupational well-being. In the existing research at home and abroad, the effect models used by psychological capital are mainly divided into four categories: main effect model, moderating effect model, dynamic effect model, and mediating effect model. The research paradigm of the main effects model is that psychological capital has a direct effect on individual and organizational outcome variables. In the field of management, a large number of studies show that psychological capital has a significant impact on the outcome variables such as employees’ job burnout, willingness to stay, resignation tendency and employees’ mental health level. In the past few years, psychological capital has been introduced into the domain of education. A large number of scholars have begun to study some related influencing factors such as psychological capital on teachers’ professional stress and professional well-being. All of them show that psychological capital has a lasting and positive influnce on teachers’ psychology and has a positive impact on teachers’ well-being.

### Anxiety

Carl Rogers (1959) proposed earlier that anxiety refers to the psychological state of uneasy and tense when there is a conflict between individual self-concept and practical experience. Sarason et al. (1960) believe that anxiety is a psychological reaction generated by individuals in the face of crisis. This emotion is often unpleasant and accompanied by physiological symptoms. Spielberger divides anxiety into trait anxiety and state anxiety, and believes that the intensity of state anxiety will fluctuate with the change of time, while trait anxiety is considered to be a relatively stable psychological trait, which reflects the individual’s perception of external stimuli as threatening emotions and behavior tendencies, as well as the possible state anxiety response to the threat.

Zhang Chunxing, a domestic scholar, pointed out that “anxiety is an emotional state intertwined by a variety of negative feelings (tension, anxiety, panic, etc.); Huang Xiting believes that anxiety is a complex emotional state such as anxiety, worried and panic when an individual is aware of the possible danger that has not yet come. Both of these descriptions emphasize the complex nature of anxiety states. Huang Xiting pointed out that the feeling of anxiety can be predictive and uneasy about things that have not yet happened. On the basis of the above research, this study defines anxiety as a natural subjective experience when an individual perceives that his own existence or existence value is threatened.

Anxiety is very common among contemporary college students. The incidence of anxiety among college students is about 10–38%, of which moderate to severe anxiety is about 5%. In the study of Zhang Fengmei and others in 2013, about 10.27% of college students’ anxiety was detected. According to rational emotional therapy, individual emotional feelings are not determined by the stimulus event itself, but are affected by the view about the stimulus event, that is, cognition and thinking model. Therefore, facing the same environmental stimulation, different people do not necessarily feel the same. For example, the anxiety experience of different individuals in the final exam is also different. Anxiety susceptible people are often introverted individuals with neurotic tendencies and unstable emotional characteristics. Research shows that excessive anxiety can be a barrier for individuals to participate in social communication, and may also hinder their best work level. For example, anxiety can seriously affect an individual’s attention. According to attention control theory, anxiety increases the individual’s attention to threat-related stimuli, thereby reducing the individual’s attention to the current cognitive task according to attention control theory.

The outbreak of SARS in China in 2003, the HINI influenza in 2009, and at the end of 2019, the outbreak of COVID-19 has brought long-term psychological impact, The impact on college students’ mental health cannot be ignored either. Through the survey results of the symptom self-rating scale SCL-90 of college students during and after the SARS epidemic, it was found that during the SARS epidemic, the measured factors (compulsion, somatization, anxiety, interpersonal relationship, depression, terror, hostility, paranoid, and psychotic) scores were higher than those after the epidemic was eliminated, and with the elimination of the epidemic, the anxiety and fear of college students were significantly relieved, but mental health problems still prevailed. During the epidemic of HINI, some college students had mental health problems of varying degrees, such as hypochondriasis, worry, anxiety and fear. Some students had a certain degree of depressive symptoms and were unable to concentrate in class. During the outbreak of COVID-19, Chang Jinghui and others conducted a survey on college students and found that 23.19% of the tested samples had mild anxiety, 2.71% had moderate anxiety, and 0.70% had severe anxiety; mild depression symptoms of college students accounted for 16.98%, moderate depression accounted for 3.17%, severe depression accounted for 1.01%. Under the pandemic situation, it is difficult for college students to study abroad. Due to the great pressure of employment competition, many students choose to take the postgraduate entrance examination, which also leads to a significant increase in admission scores compared with previous years. These problems can easily aggravate the academic anxiety of college students. The above studies show that during the outbreak of large-scale public health events and a period of time after the events, it will have varying degrees of impact on college Students’ mental health problems.

Through the previous research and analysis, the previous research on College Students’ anxiety is mainly based on College Students’ studies, employment and interpersonal relationships. Only a few studies focus on the influencing factors of large-scale public health events on college students’ anxiety state. Therefore, in the context of COVID-19’s continuous development, it is of great practical significance to analyze the impact of this incident on college students’ academic pressure, and then analyze whether it causes university students’ anxiety.

## Research Implementation

### Research Hypothesis

In order to explore the relationship between academic pressure and anxiety for college students due to changes in their learning environment and learning styles under the background of the COVID-19 pandemic, and whether positive psychological capital will have a moderating effect, ease the academic pressure of college students, and reduce anxiety levels, This paper combs the research status of college students’ academic stress, psychological capital and anxiety, analyzes the differences in demographic variables, tests the relationship between academic stress, anxiety and psychological capital, and reveals whether psychological capital can regulate academic stress and anxiety.

(1) The relationship between well-being and academic stress Andrews and Withey (1976) believed that subjective well-being (SWB) consists of two aspects: cognition and emotion. The cognitive component refers to the judgment of satisfaction with the individual’s overall life, the emotional component refers to the emotional experience in the individual’s life, which is divided into positive emotions and negative emotions. Therefore, the higher the level of satisfaction with life, the more positive emotions and less negative emotions are experienced. Wang (2015) constructed a research model of the effect of employment pressure on well-being and psychological anxiety based on the moderating effect of psychological capital when studying the stress of vocational students. The research shows that there is a significant negative correlation between well-being and employment pressure of vocational students, and psychological capital plays a moderating role in the relationship between employment pressure and well-being of vocational students. According to the previous research, when the individual’s stress perception increases, the happiness index will decrease, the positive emotion will decrease, and the negative emotion will increase; On the contrary, the individual’s happiness index increases, the positive emotion increases and the negative emotion decreases. In other words, stress is negatively correlated with happiness. Therefore, the following research hypotheses are proposed:

H1: There is a significant negative correlation between well-being and academic stress of college students.

(2) The relationship between psychological capital and anxiety

In terms of the relationship between anxiety and psychological capital, Lin et al. (2017) found that optimism and self-efficacy in psychological capital have a negative predictive effect on anxiety and depression, and resilience also has a negative predictive effect on depression. Hu and Zhu (2018) found that psychological capital of new cadets of Military Academy was significantly negatively correlated with anxiety, and psychological capital played an important mediating role in the prediction of coping style on anxiety. Liao et al. (2019) studied the effect of positive psychological capital on the anxiety of college freshmen and found that the resilience and optimism in the positive psychological capital of college freshmen affect their anxiety, and reducing the anxiety of college freshmen can improve their level of resilience and optimism. Therefore, a positive psychological capital state can effectively reduce an individual’s anxiety value. On the contrary, if the psychological capital is insufficient, the individual will show a strong anxiety perception under the influence of negative emotions such as stress. That is, psychological capital is negatively correlated with anxiety. Therefore, the following research hypotheses are proposed:

H2: The college students’ psychological capital is significantly negatively correlated with anxiety.

(3) The relationship between academic stress and anxiety

Zhang et al. (2013) believe that the psychological pressure of job selection is a tense, uneasy, strong and lasting emotional experience of college students when facing career choice, and causes corresponding physiological and behavioral reactions. Chen and Lan (2020) studied and analyzed the relationship among college students’ academic stress, psychological resilience and anxiety. The results showed that college students’ academic stress had a significant positive predictive effect on anxiety. Therefore, there is a positive correlation between an individual’s sense of stress and anxiety. If the individual cannot respond and adjust in time, the individual may experience negative emotions such as anxiety and depression. For college students, in the normalization stage of the pandemic, they are under great psychological pressure due to problems such as learning styles, practice styles, employment and postgraduate entrance examination, which will also cause serious anxiety, which may affect their physical and mental health. Therefore, the following research hypotheses are proposed:

H3: Between anxiety and academic stress of college students, there is a significant positive correlation

(4) The mediating role of psychological capital

There are few direct studies on whether psychological capital has a mediating effect on the relationship between stress and anxiety. Li (2012) research shows that college students’ well-being is directly affected by employment pressure, and psychological resilience can effectively moderate the relationship between well-being and employment pressure of college students, and play a partial mediating role. The research results of Huang and Xie (2016) show that positive psychological capital can play a partial mediating role between academic emotion and well-being. According to previous research, psychological capital may have a certain mediating effect between academic stress and well-being. Therefore, the following hypotheses are proposed:

H4: On the relationship between academic stress and anxiety of college students, psychological capital has a mediating effect.

(5) The moderating effect of psychological capital

Psychological capital refers to a positive psychological state that individuals show in the process of growth and development. Studies have shown that individuals with high psychological capital can flexibly and adaptively use multiple abilities to meet learning and work requirements, and experience a stronger sense of competence and well-being (Lu, 2004). Meng and Yang (2012) and other studies have shown that psychological capital plays a moderating role in the relationship between learning pressure, psychological anxiety and subjective well-being of college students. Therefore, individuals can effectively manage and enhance their psychological capital, which can better relieve negative emotions such as anxiety and gain a competitive advantage. High levels of psychological capital plays a moderating role in the relationship between different variables according to previous research. Therefore, the following hypotheses are proposed:

H5: On the relationship between anxiety and academic stress of college students, psychological capital has a moderating effect.

**FIGURE 1:**
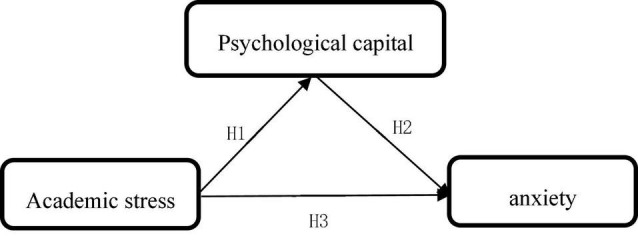
Mediation model.

**FIGURE 2:**
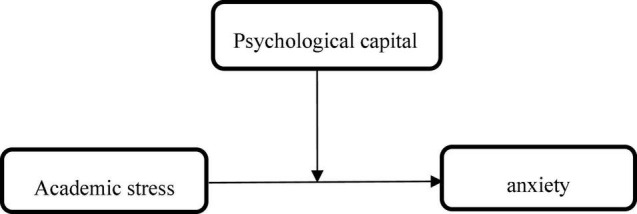
Moderation model.

According to the above research objectives and the combing of previous research, the research mediation model (see Figure 1) and moderation model (see Figure 2) are constructed. In the constructed model, the study takes college students under the background of the pandemic as the research object, analyzes and verifies the impact of academic stress on anxiety, and whether there is a mediating and moderating effect of psychological capital.

### Research Tools

The college students’ learning stress questionnaire used in this paper is a mature questionnaire adapted by Han (2017) based on “the college students’ learning stress questionnaire” compiled by Tian Lan and Deng Qi. The original scale has a total of seven dimensions, and this questionnaire selects five of them according to the actual research needs of this paper, which are five subscales of employment prospect pressure, academic achievement pressure, academic competition pressure, learning atmosphere pressure, and academic burden pressure, with a total of 24 items. Among them, 4, 8, 11, 12, and 19 are the pressure items of employment prospects; 3, 14, 15, 17, and 20 are the items of academic competition pressure; 1, 5, 10, 13, and 23 are the items of learning effect pressure; 2, 6, 7, 9 are academic atmosphere stress items; 16, 18, 21, 22, 24 are academic burden stress items. The scale adopts five grades from “completely non-conforming” to “fully conforming,” 1 for “completely non-conforming,” 2 for “relatively non-conforming,” 3 for “uncertain,” 4 for “relatively conforming,” and 5 for “fully conforming.” The higher the score, the greater the learning pressure. In this study, the Cronbach’s alpha coefficient is 0.837.

For psychological capital variables, this study adopts “the positive psychological capital questionnaire” compiled by Zhang et al. (2010), which has been relatively mature and stable in the previous research. The scale has 26 items, including self-efficacy (1, 3, 5, 7, 9, 11, 13), psychological resilience (2, 4, 6, 8, 10, 12, 14), hope (15, 17, 19, 21, 23, 25), and optimism (16, 18, 20, 22, 24, 26) 4 dimensions. The questionnaire scoring system adopts a 7-point Likert score, with 1–7, respectively indicating complete non-conforming, non-conforming, not quite conforming, unclear, relatively conforming, conforming, and completely conforming. Except that items 8, 10, 12, 14, and 25 are reverse scoring, others are positive scoring. The higher the score of each dimension, the stronger the positive psychological ability of the subjects. The total score of the dimension is the sum of the scores of the items contained. The Cronbach’s alpha coefficients of the scale and each factor were respectively 0.90, 0.86, 0.83, 0.80, and 0.76.

In this study, STAI was used to measure the anxiety of the subjects. STAI was worked out in 1970 by Spielberger et al. and revised in 1979. State anxiety subscale and trait anxiety subscale constitute the scale. Each subscale has 20 questions and a total of 40 item description questions. The State Anxiety Subscale (S-AI) is mainly used to evaluate the anxiety level of individuals under stressful situations (questions 1-20); the Trait Anxiety Subscale (T-AI) is used to evaluate the subjects’ frequent emotional experience (Questions 21-40). The scale is a Likert 4-point scale, “1” is almost none, “2” is some, “3” is often, “4” is almost always. The test-retest reliability coefficient of the scale was 0.88 for S-AI and 0.90 for T-AI.

### Research Objects and Methods

The subjects used the convenience sampling method to conduct a questionnaire survey on 280 college students in five colleges and universities in Northern Hunan in October 2021 to study whether the academic pressure has an impact on the anxiety of college students in the background of COVID-19, and whether positive psychological capital has a moderating effect on the relationship between anxiety and stress. A total of 280 questionnaires were distributed, 229 valid questionnaires were recovered, and the effective recovery rate of the questionnaires was 82%.

## Data Analysis

The data collected in this study are mainly used SPSS26.0 to analyze the recovered valid Questionnaires. In order to fully verify the reliability and validity of the questionnaire, the reliability and validity of the scale were tested, and through the difference analysis, correlation analysis, moderating effect analysis, this paper reveals the relationship between the three variables of academic stress, psychological capital and anxiety.

### Reliability Analysis of the Questionnaire

It can be seen from Table 1 that the Cronbach’s α coefficient of academic stress is 0.837, the Cronbach’s α coefficient of psychological capital is 0.901, and the Cronbach’s α coefficient of anxiety is 0.885, indicating that the reliability of each Questionnaire is good. It shows that the questionnaire has certain stability and reliability and the collected data have relatively good internal consistency.

**TABLE 1:** Reliability statistics.

variable	Cronbach’s α	Items
Academic stress	0.837	24
Psychological capital	0.901	26
anxiety	0.885	40

### Validity Analysis of the Questionnaire

Confirmatory factor analysis was carried out on the data of 229 valid questionnaires by using the CFA method and AMOS 24.0 software. The results are shown in Table 2.

**TABLE 2:** Confirmatory factor analysis results of the questionnaire (N = 287).

model	χ^2^/*df*	RMSEA	GFI	RMR	CFI
Academic stress	2.462	0.063	0.907	0.040	0.917
Psychological capital	2.884	0.071	0.921	0.041	0.914
Anxiety	2.393	0.069	0.942	0.046	0.955

It can be seen from Table 2 that in the academic stress questionnaire, the ratio of C2 to freedom is 2.462, less than 3, RMSEA = 0.063 < 0.08, GFI = 0.907 > 0.9, RMR = 0.040 < 0.05, CFI = 0.917 > 0.9; Therefore, it can be considered that the academic stress questionnaire has high structural validity. In the psychological capital questionnaire, the ratio of C2 to freedom is 2.884, less than 3, RMSEA = 0.071 < 0.08, GFI = 0.921 > 0.9, RMR = 0.041 < 0.05, CFI = 0.914 > 0.9. The psychological capital questionnaire has high structural validity. In the anxiety questionnaire, the ratio of C2 to freedom was 2.393, less than 3, RMSEA = 0.069 < 0.08, GFI = 0.942 > 0.9, RMR = 0.046 < 0.05, CFI = 0.955 > 0.9; Therefore, the anxiety questionnaire has high structural validity.

### Descriptive Statistical Analysis of Demographic Variables

According to Table 3, in terms of gender distribution, there are 124 females, accounting for 54.1%, and 105 males, accounting for 45.9%. In terms of grade distribution, there are 6 freshmen, accounting for 2.6%, 71 sophomores, accounting for 31.0%, and 62 juniors, accounting for 27.1%. There are 90 seniors, accounting for 39.3%. In terms of the distribution of student leaders, there are 125 people who are yes, accounting for 54.6%, and 104 people who are not, accounting for 45.4%. In terms of the distribution of scholarships, there are 126 people with yes, accounting for 55.0%, and 103 people without it, accounting for 45.0%. In terms of the distribution of parents’ marital status, 175 are married, accounting for 76.4%, and 54 are divorced, accounting for 23.6%. In terms of the distribution of only child, there are 94 people who are yes, accounting for 41.0%, and 135 people who are not, accounting for 59.0%.

**TABLE 3:** Descriptive statistical analysis results.

Variable	Category	Count	Percentage
Gender	Male	105	45.9
	Female	124	54.1
Grades	Freshman	6	2.6
	Sophomore	71	31.0
	Junior	62	27.1
	Senior	90	39.3
Student leaders	Yes	125	54.6
	No	104	45.4
Scholarship	Yes	126	55.0
	No	103	45.0
Marital status of parents	Married	175	76.4
	Divorced	54	23.6
Only child	Yes	94	41.0
	No	135	59.0

### Difference Analysis

(1) Gender difference analysis results

It can be seen from Table 4 that the difference in academic stress between different genders is statistically significant (*p* < 0.05), and the results indicate that the academic pressure of boys is significantly lower than that of girls. Analysis of the reasons, this may be related to the different spare time habits of boys and girls in college life. The daily sports activities and video games that male college students participate in have a good stress relief function, which may lead to the relatively low academic pressure of male students. The differences in psychological capital between different genders are not statistically significant (*p* > 0.05). The difference in anxiety between different genders is not statistically significant (*p* > 0.05).

**TABLE 4:** Difference test results of different genders on academic stress, psychological capital, and anxiety.

Dimension	Gender	Number of cases	Average value	Standard deviation	t	*p*
Academic stress	Male	105	4.37	0.49	−2.216	0.028
	Female	124	4.49	0.36		
Psychological capital	Male	105	2.45	0.85	1.913	0.057
	Female	124	2.25	0.78		
Anxiety	Male	105	3.18	0.43	−1.230	0.220
	Female	124	3.25	0.44		

(2) Analysis results of grade differences

From Table 5, it can be seen that the difference in academic pressure of different grades is statistically significant (*p* < 0.001). Multiple comparison results show that the academic pressure of senior students is significantly higher than that of juniors, sophomores and freshmen. The difference in psychological capital of different grades is statistically significant (*p* < 0.01). Multiple comparison results showed that the psychological capital of junior and sophomore students is significantly higher than that of freshmen and seniors. The difference in anxiety among different grades is statistically significant (*p* < 0.001). The multiple comparison results showed that the anxiety of seniors is significantly higher than that of juniors, sophomores, and freshmen. Senior students are bound to have high anxiety because of the pressure of postgraduate entrance examination and employment. Students who plan to take postgraduate entrance exams, study abroad, and take exams for civil servants experience a sharp increase in academic pressure. However, college students in other grades have not yet faced these pressures, so the values of stress and anxiety are significantly lower than those of senior students. As senior students are affected by the above analysis reasons, freshmen have entered a new environment, and sophomores and juniors have adapted to college life. Therefore, in terms of psychological capital, freshmen and juniors are significantly lower than sophomores and juniors.

**TABLE 5:** Difference test results of academic stress, psychological capital, and anxiety in different grades.

Dimension	Grades	Number of cases	Average value	Standard deviation	F	*p*	Turkey
Academic stress	Freshman	6	4.10	0.62	9.065	<0.001	Senior > Junior, sophomore, freshman
	Sophomore	71	4.28	0.52			
	Junior	62	4.41	0.36			
	Senior	90	4.59	0.30			
Psychological capital	Freshman	6	2.73	1.14	12.422	<0.001	Junior, sophomore > Senior
	Sophomore	71	2.69	0.85			
	Junior	62	2.43	0.70			
	Senior	90	1.98	0.70			
Anxiety	Freshman	6	2.82	0.33	11.228	<0.001	Senior > Junior, sophomore, freshman
	Sophomore	71	3.06	0.41			
	Junior	62	3.19	0.40			
	Senior	90	3.40	0.42			

(3) Difference analysis results of whether one is a student leader

It can be seen from Table 6 that the difference in academic stress is not statistically significant whether one is a student leader or not (*p* > 0.05). the difference in psychological capital is not statistically significant whether one is a student leader or not (*p* > 0.05). Whether one is a student leader, the difference in anxiety is not statistically significant (*p* > 0.05). The data analysis shows that whether they are student leaders, the academic pressure and anxiety state they face are the same as those of other students. In the same learning resources and learning environment, the situation of psychological capital is also similar. Therefore, the perception of academic pressure, psychological capital and anxiety of student leaders and other students is consistent.

**TABLE 6:** Is the difference test result of whether one is a student leader on academic stress, psychological capital, and anxiety.

Dimension	Student leader	Number of cases	Average value	Standard deviation	t	*p*
Academic stress	Yes	125	4.42	0.47	−0.363	0.717
	No	104	4.44	0.38		
Psychological capita	Yes	125	2.33	0.84	−0.236	0.814
	No	104	2.36	0.79		
Anxiety	Yes	125	3.23	0.47	0.331	0.741
	No	104	3.21	0.41		

(4) Difference analysis results of whether to obtain scholarship

From Table 7, it can be seen that whether you have won a scholarship or not, the difference in academic stress is no statistically significant (*p* > 0.05). The difference in psychological capital between those who received scholarships or not is no statistically significant (*p* > 0.05). The difference in anxiety between those who received a scholarship or not is no statistically significant (*p* > 0.05).

**TABLE 7:** Test results of differences in academic stress, psychological capital and anxiety whether you have won a scholarship.

Dimension	Scholarship	Number of cases	Average value	Standard deviation	t	*p*
Academic stress	yes	126	4.43	0.47	−0.239	0.811
	no	103	4.44	0.38		
Psychological capita	yes	126	2.36	0.85	0.457	0.648
	no	103	2.31	0.78		
Anxiety	yes	126	3.21	0.46	−0.551	0.582
	no	103	3.24	0.41		

(5) Analysis results of differences in parents’ marital status

From Table 8 it can be seen that the difference in academic stress of college students with different parents’ marital status is not statistically significant (*p* > 0.05). The difference in psychological capital with different parents’ marital status is no statistically significant (*p* > 0.05). The difference in anxiety with different parents’ marital status is no statistically significant (*p* > 0.05). This result may be related to the current phenomenon of high divorce rate, the increase in the number of divorced families, the change of social concepts, the enhancement of students’ independence, and the influence of parents’ marital status on college students has become smaller and smaller, with no significant difference.

**TABLE 8:** Difference test results of parents’ marital status on academic stress, psychological capital, and anxiety.

dimension	Marital status of parents	number of cases	average value	standard deviation	t	p
Academic stress	married	175	4.43	0.46	−0.057	.955
	divorced	54	4.44	0.32		
Psychological capita	married	175	2.35	0.83	0.269	0.788
	divorced	54	2.32	0.77		
anxiety	married	175	3.21	0.46	−0.464	0.643
	divorced	54	3.24	0.37		

(6) Difference analysis results of whether one is an only child

From Table 9 it can be seen that the difference in academic pressure between college students who are only children or not is a statistically significant (*p* < 0.01). The results suggest that the academic pressure of only children is significantly higher than that of non-only children. The difference in psychological capital of who are only children or not is a statistically significant (*p* < 0.001), and the results suggest that the psychological capital of the only child is significantly lower than that of the non-only child. The difference in anxiety among different only children is a statistically significant (*p* < 0.001), and the results suggested that the anxiety of only children was significantly higher than that of non-only children.

**TABLE 9:** Is the difference test result on academic stress, psychological capital and anxiety of whether an only child.

Dimension	Only child	Number of cases	Average value	Standard deviation	t	*p*
Academic stress	Yes	94	4.53	0.30	3.261	0.001
	No	135	4.36	0.49		
Psychological capita	Yes	94	2.11	0.70	−3.751	<0.001
	No	135	2.51	0.85		
Snxiety	Yes	94	3.35	0.39	3.974	<0.001
	No	135	3.13	0.45		

### Correlation Analysis

Table 10 shows that there is a significant positive correlation between academic stress and anxiety, and the correlation coefficient is 0.370; and a significant negative correlation with psychological capital, and the correlation coefficient is −0.893; Between psychological capital and anxiety, there is a significant negative correlation, and the correlation coefficient is −0.557. Among the three variables, there is a significant correlation between pairs. There is a significant negative correlation between academic pressure and psychological capital, and there is a significant positive correlation between academic pressure and anxiety, indicating that the greater the academic pressure, the worse the performance of psychological capital and the higher the anxiety value. There is a significant negative correlation between psychological capital and anxiety, indicating that the better the performance of psychological capital, the lower the perceived anxiety value.

**TABLE 10:** Correlation analysis results between academic stress, psychological capital and anxiety.

		Academic stress	Psychological capita	anxiety
Academic stress	Pearson correlation Significance (two-tailed)	1	−0.893**	0.370**
			0.000	0.000
	Number of cases	229	229	229
Psychological capita	Pearson correlation Significance (two-tailed)	−0.893**	1	−0.557**
		0.000		0.000
	Number of cases	229	229	229
anxiety	Pearson correlation Significance (two-tailed)	0.370**	−0.557**	1
		0.000	0.000	
	Number of cases	229	229	229

**The correlation is significant at the 0.01 level (two-tailed).

### Analysis of Mediating Effect

From Table 11, it can be seen from the above table that academic stress can significantly positively predict anxiety (β = 0.37, t = 6.000, *p* < 0.001), academic stress can significantly negatively predict psychological capital (β = −0.893, t = −29.926, *p* < 0.001), psychological capital can significantly negatively predict anxiety (β = −0.602, t = −9.702, *p* < 0.001), psychological capital can significantly negatively predict anxiety (β = −0.557,t = −10.104, *p* < 0.001), Academic stress can significantly positively predict anxiety (β = 0.247, t = 5.462, *p* < 0.001), which means that between academic stress and anxiety, psychological capital plays a partial mediating role. Since it is a partial mediating effect, the proportion of the mediating effect is 45.29% calculated through the calculation formula of effect proportion.

**TABLE 11:** Analysis of the mediating effect of psychological capital on academic stress, and anxiety.

Step	Regression equation	R2value	SE value	B value	T value	*p* value
Step 1	Y = 0.370X	0.137	0.063	0.370	6.000	0.005
Step 2	M = 0.893X	0.798	0.067	0.893	29.926	0.005
Step 3	Y = −0.602M + 0.247X	0.391	0.118	−0.602	−9.702	0.005
			0.062	0.247	5.462	0.005

### Moderating Analysis

Table 12 shows that the interaction between psychological capital and academic stress can significantly negatively predict anxiety (β = −0.15, t = −4.51, *p* < 0.001), so between academic stress and anxiety, psychological capital plays a partial moderating role.

**TABLE 12:** Test results of the moderating effect of psychological capital on academic stress, and anxiety.

Variable	Coefficient	Standard error	t	*p*
Constant	3.17	0.02	131.07	<0.001
Academic stress	−0.23	0.15	−1.59	0.112
Psychological capita	−0.49	0.06	−7.54	<0.001
Academic stress × psychological capital	−0.15	0.03	−4.51	<0.001

*Dependent variable: anxiety, F = 59.23, p < 0.001.*

## Conclusion

The results of this study show that the differences in academic stress between different genders is statistically significant. The results suggest that the academic stress of boys is significantly lower than that of girls, indicating that girls have a high degree of academic pressure perception, at the same time, because boys often participate in playing ball, fitness, video games and other activities, they can also alleviate the pressure to varying degrees, suggesting that college students of different genders may have great differences in the ways of stress relief. In the two variables of psychological capital and anxiety, the differences between different genders are not statistically significant. According to the results of multiple comparison analysis, the academic stress and anxiety of seniors were significantly higher than those of juniors, sophomores and freshmen. Analyzing the reasons, senior students face the pressure of postgraduate entrance examination, civil service examination and employment, especially in the past two years, due to the impact of the pandemic, the difficulty of employment has increased and the number of people taking the postgraduate entrance examination has increased. For senior students, the academic pressure and anxiety perception will be significantly improved. Multiple comparison results suggest that junior and sophomore students have higher psychological capital, suggesting that sophomore and junior students have well adapted to college life, and do not need to face the pressure of employment and graduation. Therefore, the situation of positive psychological capital is significantly higher than that of freshmen and seniors. According to the results of multiple analysis, in the three variables of academic stress, psychological capital and anxiety, the differences in the factors of whether you are a student leader and whether you have received a scholarship are not statistically significant.

The results of this study show that there is a significant correlation between psychological capital, academic stress and anxiety. There is a significant negative correlation between psychological capital and academic stress. This result shows that the higher the perception of academic stress, the more it will affect psychological capital and lead to a decline in positive psychological capital, compared with previous research results, this result is consistent with it (Wang, 2015). Between academic stress and anxiety, there is a significant positive correlation, indicating that the greater the academic stress, the more likely it is to lead to anxiety, compared with previous research results, this result is consistent with it (Liao et al., 2019). This also suggests that we should focus on the college students’ academic stress. Especially in the context of normalization of COVID-19 pneumonia, the uncertainty of class forms and the reform of teaching methods, as well as the tasks and pressures of original academic and employment, It is necessary to propose effective ways and methods to relieve stress, reduce anxiety caused by stress, and effectively improve the mental health of college students. According to the research results, between psychological capital and anxiety, there is a significant negative correlation, psychological capital plays a partial moderating role between academic stress and anxiety, and psychological capital plays a partial mediating role between academic stress and anxiety, and. The results of this study fully demonstrate that positive psychological capital can effectively reduce anxiety caused by academic pressure. As a positive psychological resource, psychological capital can fully mobilize positive energy and effectively resist external setbacks. It can play an important role in alleviating college students’ academic pressure and reducing anxiety. This result suggests that the college students positive psychological capital status of should be improved, which can effectively relieve stress and reduce anxiety. This is also consistent with previous research findings (Chen and Lan, 2020).

According to the above analysis results, this study believes that as college students, in the context of the normalization of the pandemic, they should actively participate in the activities and learning related to mental health organized by the school, objectively determine their learning goals and career planning, identify with their self-worth, maintain good interpersonal relations with their classmates, and enhance their adaptability to the normalization environment of the pandemic. From the perspective of colleges and universities, the adaptability differences of college students to changes in learning environment and learning methods should be considered, and good channels and platforms for emotional relief should be provided for students through some psychological lectures and the establishment of psychological counseling hotlines. Schools should also do a good job in the continuous publicity and daily implementation of pandemic prevention knowledge. Strengthen the cultivation of students’ independent thinking ability and discrimination ability, and timely issue clear official notice interpretation.

This study investigates the relationship between academic stress and anxiety among college students in the context of the normalization of the pandemic, which is different from previous discussions on related issues in the context of epidemic outbreaks. The research on the impact of coronary pneumonia on mental health is also mainly focused on medical staff, patients and their families, and there is a lack of research on the group of college students. As for the influencing factors of college students’ academic stress, previous studies mostly start with specific methods of stress relief, but few studies discuss it from the perspective of the moderating effect of college students’ psychological capital. Therefore, the research perspective and research content are innovative to a certain extent.

This study still has some certain limitations: (1) In terms of sample selection, the research object of this study is college students in five colleges and universities in Northern Hunan. Due to the differences in the level of social and economic development with other regions in the country, the promotion scope of the research results will be limited, and the future research samples can be further expanded to other types of schools in other provinces and (2) This study only discusses the relationship between psychological capital, academic stress and anxiety of college students, and the relationship between different dimensions of influencing factors and anxiety can be further explored in the future.

## Data Availability Statement

The original contributions presented in the study are included in the article/supplementary material, further inquiries can be directed to the corresponding author.

## Author Contributions

YY: writing-original draft. PY: supervision. Both authors contributed to the article and approved the submitted version.

## Conflict of Interest

The authors declare that the research was conducted in the absence of any commercial or financial relationships that could be construed as a potential conflict of interest.

## Publisher’s Note

All claims expressed in this article are solely those of the authors and do not necessarily represent those of their affiliated organizations, or those of the publisher, the editors and the reviewers. Any product that may be evaluated in this article, or claim that may be made by its manufacturer, is not guaranteed or endorsed by the publisher.
